# Functional roles and regulatory mechanisms of paeonol in the treatment of liver disease

**DOI:** 10.1007/s13659-025-00554-3

**Published:** 2026-01-06

**Authors:** Min Shu, Mengzhou Fang, Tao Xu, Qi Yan

**Affiliations:** 1 Inflammation and Immune Mediated Diseases Laboratory of Anhui Province, School of Pharmaceutical Sciences, Anhui Medical University, Hefei, 230032 China; 2 Anhui Key Lab of Bioactivity of Natural Products, Institute for Liver Diseases of Anhui Medical University, Anhui Medical University, Hefei, 230032 China; 3 School of Medical Imaging, Bengbu Medical University, Bengbu, 233030 China; 4 Department of Clinical Laboratory, the Second Affiliated Hospital of Anhui Medical University, Hefei, 230032 China

**Keywords:** Paeonol, Liver diseases, Anti-inflammatory, Signaling pathways, Drug delivery systems

## Abstract

Liver disease is a serious threat to human health, so its prevention and treatment have been the focus of medical research. In the past few years, natural products have proved to be promising and valuable in the treatment of liver diseases. The bioactive substance paeonol, extracted from the root bark of peony (Paeonia lactiflora) of the buttercup family, is a promising drug candidate because of its low toxicity and multifaceted pharmacological properties. This review comprehensively explored the therapeutic potential of paeonol in different liver pathologies as well as the regulatory mechanisms. Despite its promising potential, the poor solubility and rapid metabolism of paeonol hindered its clinical translation. To improve bioavailability and liver targeting, we highlighted the potential of paeonol as a next-generation therapy for liver diseases by integrating preclinical evidence and technological advances, while exploring key avenues for future research, such as metabolic regulation and smart nanocarrier design.

**Graphical abstract:**

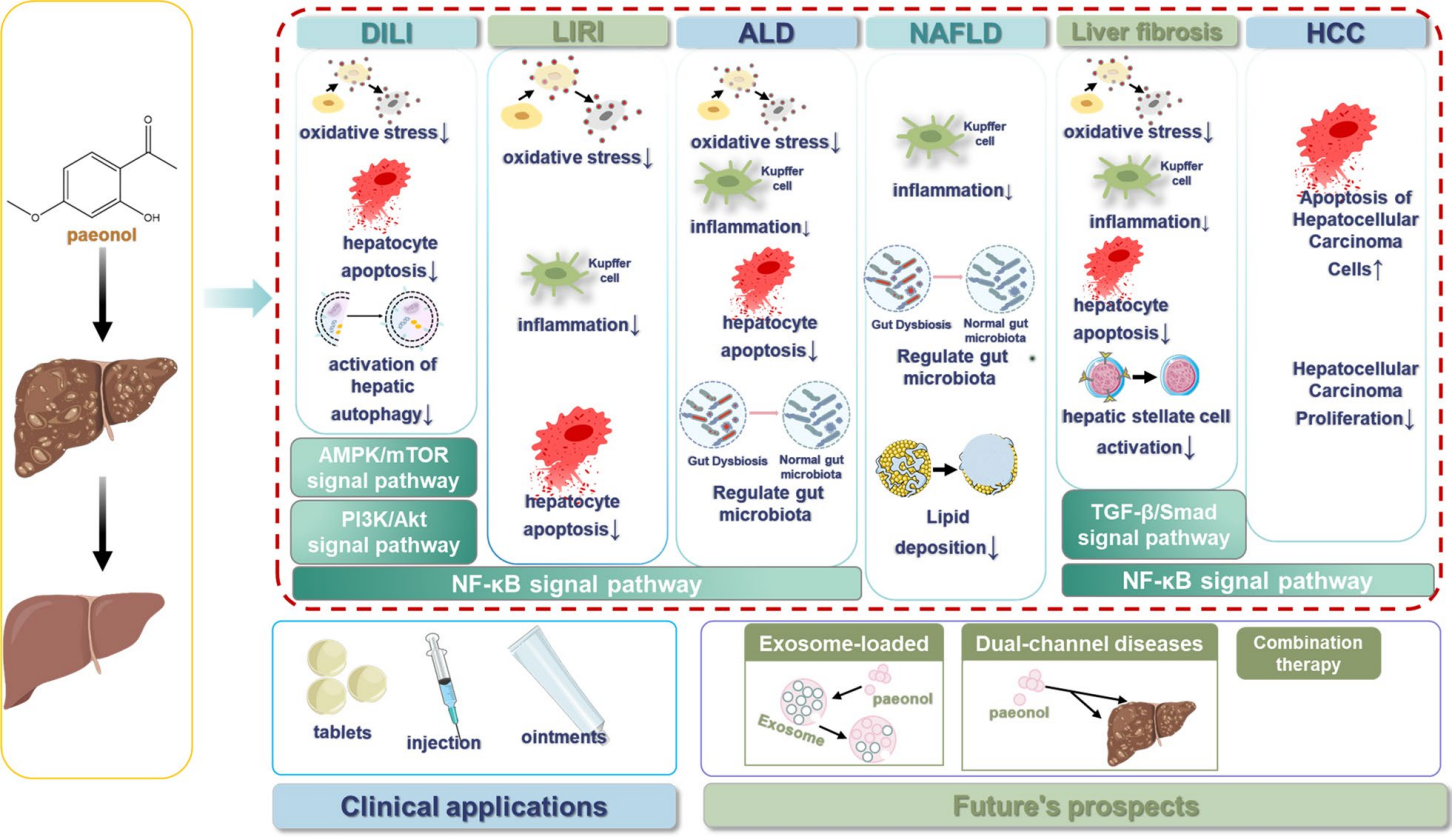

## Introduction

Liver diseases can be categorized into acute liver injury (ALI), viral hepatitis, alcoholic liver disease (ALD), metabolic dysfunction-associated fatty liver disease (MAFLD), liver fibrosis, and hepatocellular carcinoma (HCC) depending on the etiology and pathogenesis [1]. The global incidence of liver disease is on the rise, with increasing risks of developing the condition. Annually, approximately 2 million people die from liver disease, accounting for 4% of global mortality [2]. Among these, HCC, the end-stage manifestation of liver disease, caused around 830,000 deaths in 2020, equivalent to 8.3% of cancer-related deaths worldwide [3]. Liver diseases are caused by numerous factors, including drugs, chemicals, viral infections, excessive consumption of alcohol, dystrophy, and disorders of acid-base balance [4, 5]. Fortunately, increasing attention has been directed toward the use of highly effective and low-toxicity components from natural products for the treatment of liver disease, and some progress has been made.

Among them, the pharmacological mechanism of paeonol, an active monomer component derived from the root bark of peony (Paeonia lactiflora) of the buttercup family, has attracted much attention in recent years as a natural compound with therapeutic potential for liver disease. According to previous studies, paeonol exhibits broad-spectrum bioactivities through multi-target mechanisms, including anti-inflammatory [6, 7], anti-tumor [8, 9], anti-fibrotic [10], and nephroprotective [11]effects through various mechanisms. Of particular note, a series of breakthroughs have been made in the study of paeonol in the therapeutic and prophylactic efficacy against liver diseases. In a CCl_4_-induced liver fibrosis model, paeonol significantly improved the oxidative stress and inflammatory microenvironment by inhibiting hepatic stellate cells (HSCs) activation [12]. In addition, paeonol has been shown to attenuate lipopolysaccharide (LPS)-induced hepatocyte damage by improving mitochondrial dysfunction, reducing peroxide production, and decreasing hepatocyte apoptosis [13]. Therefore, a deeper understanding of paeonol may bring new light to the therapy of liver diseases.

In view of the multidimensional regulatory role of paeonol in the pathological process of liver disease, this review systematically summarized the progress of its mechanisms of action in different liver disease models, focusing on the core protective pathways of paeonol against liver injury, the regulatory characteristics of key signaling networks and the potential translational medical value. By integrating the existing research results, we provided a theoretical basis for further development of new strategies based on paeonol for the treatment of liver diseases.

## Physicochemical properties and bioavailability of paeonol

**Fig. 1:**
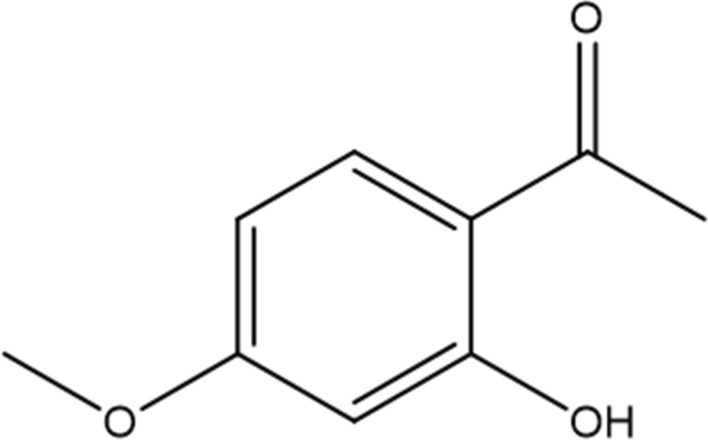
Chemical structure of paeonol (2′-hydroxy-4′-methoxyacetophenone). The phenolic hydroxyl and methoxy groups contribute to its protein-binding capacity

Paeonol (chemical name: 2′-hydroxy-4′-methoxyacetophenone) (Fig. 1) is a white crystalline compound containing phenolic hydroxyl and methoxy groups. Its physicochemical properties show typical polyphenolic characteristics, exhibiting negligible aqueous solubility but pronounced miscibility in polar organic solvents such as ethanol [14]. In addition, the phenolic hydroxyl groups in the molecular structure of paeonol endow it with significant protein binding capacity. Studies have shown that paeonol can form complexes with a variety of proteins through hydrogen bonding and hydrophobic interactions [14, 15]. Thermodynamic analysis shows that the binding process could be described as spontaneous (ΔG < 0) and exothermic (ΔH < 0), with hydrogen bonding and van der Waals forces dominating [15]. Meanwhile paeonol, as a reversible hybrid tyrosinase inhibitor, which exhibited a dose-dependent inhibitory effect (IC50 = 98.3 ± 1.3 μM), bound to tyrosinase via a static burst mechanism with a binding constant of Ka = 0.16 × 10^4^–0.39 × 10^4^ L/mol and a binding site number of n ≈ 0.74–0.82[15]. In food systems, paeonol retarded browning by a dual mechanism: (i) inhibiting polyphenol oxidase (PPO) and peroxidase (POD) activities; and (ii) enhancing ascorbate peroxidase (APX) activity [15]. Notably, the bioactivity of paeonol was significantly affected by environmental factors, with temperature, light and oxygen exposure all accelerating degradation [15]. Surface plasmon resonance analysis showed significant differences in the binding strength of paeonol to different proteins: strong binding to β-lactoglobulin (lgKa = 6.75), bovine serum albumin (lgKa = 6.28), etc., and weak binding to α-lactalbumin (lgKa = 3.92), lactoferrin (lgKa = 4.54) [14]. This selective binding property cannot be dissociated from vivo distribution and metabolic pathway.

Pharmacokinetics are critical in determining the safety and efficacy of a drug and are important in guiding the clinical use of paeonol. The oral bioavailability of paeonol has been reported to be low, primarily due to its rapid metabolism and clearance, largely attributable to first-pass metabolism [16]. In addition, in animal models, paeonol is rapidly absorbed into the blood circulation by the intestine and rapidly distributed to multiple organs after oral administration, showing rapid absorption-distribution-elimination characteristics [16]. This is attributed to its short time to peak (T_max_) and half-life (T_1/2_). Notably, intranasal administration greatly improved bioavailability (52.37%), and acidic environment (pH4) and hypertonic conditions (1.8% NaCl) further improved absorption efficiency [17]. These findings suggested that the development of novel delivery systems may be an effective strategy to improve liver targeting.

## Overview of liver disease

Liver diseases are caused by viruses, ethanol, drugs, and autoimmune abnormalities. Chronic liver diseases primarily include viral hepatitis B and C (HBV and HCV), ALD, non-alcoholic fatty liver disease (NAFLD). These conditions may progress to non-alcoholic steatohepatitis, liver fibrosis, and ultimately lead to end-stage liver diseases such as cirrhosis or HCC [18]. Acute liver diseases, which typically include acute liver failure, LIRI, and acute alcoholic hepatitis, are characterized by sudden onset, rapid progression and high lethality [19]. Globally, liver disease ranks 11th among causes of death, more than 800 million people are living with liver disease, and it is estimated to cause around 2 million deaths each year, accounting for 4% of all causes of death [20]. Approximately two-thirds of disease-related deaths occur in men, with cirrhosis-related complications and HCC being the main causes, whereas acute hepatitis contributes to a smaller proportion [20]. The high mortality rates of cirrhosis and HCC have led us to emphasize the ability to prevent and treat liver diseases.

Liver disease is often caused by cellular oxidative stress and inflammatory reactions. Following hepatocellular injury, cytokines and proteins that initiate an inflammatory response rapidly increase, leading to excessive production of extracellular matrix (ECM). The accumulation of ECM subsequently disrupts the normal liver architecture, resulting in liver dysfunction, followed by fibrosis, cirrhosis, and HCC [21, 22]. Currently, the primary clinical approach to treating liver disease is medical therapy, including hepatoprotective and enzyme-lowering drugs (bicyclol and biphenyl dibenzoate), cholestasis-relieving agenets (ursodeoxycholic acid and butanedisulfonic acid adenosylmethionine), and detoxifying agents (reduced glutathione and prohibition). Surgical approaches are generally limited to early-stage HCC, as they are often ineffective and associated with a high recurrence rate. In view of the significant health and social influence of liver diseases, higher demands have been placed on their treatment. Among them, natural products are often characterized by novel structures with high activity and few adverse effects, making the development of natural products imperative in the clinical management of liver diseases.

## The functional role of paeonol in liver disease

The progression of liver disease typically follows a sequential pattern from acute injury to chronic inflammation, fibrosis, and ultimately carcinogenesis. Paeonol exerts protective effects at multiple stages of this process, primarily through its anti-inflammatory, antioxidant, and anti-fibrotic activities. To systematically evaluate the therapeutic potential of paeonol therapeutic potential, this review analyzed its pharmacological actions against disease progression at each pathological stage and associated complications (Table 1).

**Table 1:** Functional overview of paeonol in liver disease

Medicine	Disease type	Models	Route of administration	Drug delivery dose	Functions	References
Paeonol	Drug-induced liver injury	Epirubicin(EPI)-injected 4T1 tumor bearing mice	gavage (stomach filling)	20 mg/kg	Inhibition of oxidative stress and hepatocyte apoptosis in the liver which caused by EPI	[28]
	Drug-induced liver injury	EPI-injected breast tumour-bearing mice	gavage (stomach filling)	30 mg/kg	Attenuating EPI-induced activation of autophagy in the liver	[29]
	Drug-induced liver injury	Lipopolysaccharide (LPS)-treated L02 human normal hepatocytes	cell incubation	0.1, 1.0, 10.0, 100.0 μg/ml	Attenuating LPS-induced liver injury	[13]
	Drug-induced liver injury	Male Wistar rats injected with Methotrexate (MTX)	oral (administered by mouth)	100 mg/kg	Attenuates MTX-induced oxidative stress in the liver and hepatocyte death	[34]
	Liver ischemia/reperfusion injury	Liver ischemia/reperfusion injury in male Wistar rats	oral (administered by mouth)	100 mg/kg	Suppressing oxidative stress, inflammatory response, and hepatocyte apoptosis in the liver	[54]
	Alcoholic liver disease	Alcohol-induced liver injury models in Kunming male mice	oral (administered by mouth)	100, 200, 400 mg/kg	Reducing alcohol-induced oxidative stress, inflammation, lipogenesis, and hepatocyte apoptosis in the liver	[58]
	Alcoholic liver disease	Modeling of liver injury in male C57BL/6 mice with alcohol	oral (administered by mouth)	25, 100 mg/kg	Reducing oxidative stress and inflammatory response in the liver	[59]
	Alcoholic liver disease	Modeling of liver injury in male C57BL/6 mice with alcohol	oral (administered by mouth)	120, 240, 480 mg/kg	Alleviating alcohol-induced intestinal barrier dysfunction and acute inflammatory injury in the liver	[67]
	Non-alcoholic fatty liver disease	Male C57BL/6 mice fed with methionine choline (MCD) high-fat diets	oral (administered by mouth)	100, 200 mg/kg	Improvement of intestinal mucosal barrier and reduction of liver inflammation in non-alcoholic steatohepatitis (NASH) mice	[72]
	Liver fibrosis	CCl_4_-administered male Sprague–Dawley rats	intraperitoneal injection	50, 100, 200 mg/kg	Improvement of steatosis status, reduction of bridging fibrotic septum thickness and induction of mitochondrial apoptosis in hepatic stellate cells	[83]
	Liver fibrosis	CCl_4_-administered male C57BL/6 J mice	oral (administered by mouth)	100 mg/kg	Inhibition of oxidative stress and inflammation in the liver, and hematopoietic stem cell activation	[12]
	Liver fibrosis	Male Sprague–Dawley rats fed with olive oil CCl_4_	gavage (stomach filling)	100 mg/kg	Inhibiting collagen production and inducting apoptosis in hepatic stellate cells	[84]
	Hepatocellular carcinoma	Tumor-bearing mice model	intraperitoneal injection	100, 200, 400 mg/kg	Induction of apoptosis in tumor cells	[91]
	Hepatocellular carcinoma	Human hepatoma cells HepG2 and SMMC7721	cell incubation	15.63, 31.25, 62.5 mg/L	Inhibition of proliferation and induction of apoptosis in hepatocellular carcinoma cells	[92]
	Hepatocellular carcinoma	Human hepatocellular carcinoma cell lines BEL-7404 and SMMC-7721	cell incubation	0, 2.6, 5.19, 10.39, 20.78, 41.55, 83.09, 166.18 mg/L	Induction of apoptosis in hepatocellular carcinoma cells	[93]
	Hepatocellular carcinoma	Hepatocellular carcinomacell lines (Hep3B and Huh-7)	cell incubation	0, 50, 100, 200, 400, 800 μM	Promoting apoptosis in hepatocellular carcinoma cells	[100]
	Hepatocellular carcinoma	Paraffin-embedded cancerous and noncancerous adjacent tissues	cell incubation	47, 94, 188, 376 μM(10 mg/mL)	Promoting apoptosis in hepatocellular carcinoma cells	[104]

### Drug-induced liver injury

Drug-induced liver injury (DILI) is defined as hepatotoxicity caused by various chemicals, biologics, and other drugs, and is one of the most common adverse drug reactions in clinical practice [23]. The prevalence of DILI is estimated to be about 14–19 cases/100,000 people [24], among them 13.00% developing chronic DILI, 44.40% developing hepatocellular DILI, and 1.08% developing liver failure, and the mortality rate is estimated at 0.39% [25]. The current treatment for DILI is to discontinue the offending medication as quickly as possible after the diagnosis is clarified[26]. As the utilization of natural products in liver disease, scientists are gradually discovering the important role of paeonol in DILI.

The cholestatic DILI phenotype is characterized by impaired bile secretion and bile reflux, resulting in elevated serum alkaline phosphatase (ALP) and glutamate transaminase (GGT) levels [27]. Consistent with the empirical findings of Wu et al. paeonol demonstrated significant attenuation of Epirubicin (EPI)-provoked hepatocellular oxidative stress and pro-inflammatory cascades, thereby effectively forestalling the pathogenesis of drug-induced hepatotoxicity (DILI). As demonstrated in the study of Wu et al., paeonol inhibited Epirubicin (EPI)-induced elevated levels of oxidative stress and inflammation in the liver, thereby effectively forestall the pathogenesis of DILI, and was manifested by reduced levels of ALP and GGT in the blood of EPI-administered mice [28]. Not coincidentally, Xu et al. further demonstrated the protective effect of paeonol against EPI-induced severe liver tissue injury in mice from a metabolomics perspective [29]. Combined with the results reported in the existing literature, i.e., hepatocellular injury and necrosis can cause impaired protein synthesis and protein catabolism in the liver, which leads to elevated levels of free amino acids in both serum and liver [30, 31]. The researchers also screened seven endogenous metabolites as potential biomarker candidates for EPI-induced liver injury. They found that serum oleic acid and glycine concentrations in EPI-treated mice were lower than in normal mice, while succinate, malate, L-valine, L-leucine, and serine concentrations were higher. However, paeonol treatment was able to reverse this change brought on by EPI and bring it closer to normal levels.

Methotrexate (MTX) serves as a chemotherapeutic drug to treat various autoimmune diseases and malignant tumors [32, 33]. Unfortunately, the use of MTX is invariably associated with multiorgan toxicity, with liver impairment emerging as the predominant manifestation [32]. Researchers have also found that paeonol is effective in preventing MTX-induced hepatotoxicity by inhibiting oxidative stress, fibrosis, and apoptosis in the liver [34]. Beyond its effects on oxidative stress and inflammation, paeonol has been demonstrated to significantly augment the expression of the drug efflux transporter proteins P-glycoprotein (P-gp) and multidrug resistance-associated protein 2 (MRP-2). P-gp protects the organ from endogenous and exogenous toxins by excreting toxic compounds including chemotherapeutic and other drugs[35]. In contrast, at the hepatocyte brush border, the integration of ATP-dependent transporter proteins, particularly MRP-2, connects this distinctive membrane morphology with its function [36, 37]. The dislocation of MRP-2 from the canalicular membrane, with structural alterations of the toothbrush-like rim, is a hallmark of cholestatic liver disease [38, 39]. This mechanism adds another layer of protection of paeonol to the liver, thus preventing endogenous and exogenous toxin-induced acute liver injury. Therefore, studying the role of paeonol in DILI is important for the treatment of various DILIs.

Reactive oxygen species (ROS) are primarily derived from mitochondria, and an imbalance in their production will results in diminished transmembrane potential across mitochondrial membranes [40]. And ROS have a crucial role in cellular damage [41, 42]. Malondialdehyde (MDA) is the end substance of lipid peroxidation. Superoxide dismutase (SOD) constitutes a crucial endogenous antioxidative enzyme that is highly expressed in the liver and scavenges harmful ROS [43, 44]. XU et al. also found that paeonol reduced the elevated levels of MDA and the production of ROS in mice induced by lipopolysaccharides (LPS) and simultaneously increased the LPS-induced SOD levels, which also exerted hepatoprotective properties against LPS-induced liver injury [13]. At the same time, depolarization of mitochondria leads to the release of transmembrane proteins, which ultimately leads to the activation of caspase-3 [45, 46]. When caspase-3 is activated, DNA breakage, nucleosome condensation and apoptosis occur [47]. XU et al. also found that paeonol reversed the LPS-induced rise in cleaved-caspase-3 and enhanced the LPS-induced fall in mitochondrial transmembrane potential. These results further demonstrated that paeonol could improve mitochondrial dysfunction, reduce superoxide production, and decrease hepatocyte apoptosis, thus attenuating LPS-induced hepatocyte injury.

### Liver ischemia–reperfusion injury

Liver ischemia/reperfusion (I/R) injury frequently occurs after liver transplantation and liver resection. By synthesizing information from multiple clinical studies and basic research, it is now believed that LIRI is connected with a series of complex events, including the dysfunction of mitochondria and oxidative stress disorders, and additional pathological phenomena that are yet to be defined [48]. LIRI can lead to early post-transplantation graft dysfunction (EAD), primary nonfunction (PNF), and even acute-phase graft loss, which in turn affects patient prognosis [49]. Fortunately, the emergence of the natural product paeonol has brought a new light to the treatment of LIRI.

Heme oxygenase-1 (HO-1) could protect hepatocytes [50, 51], and hippocampal neurons [52, 53] from ischemia–reperfusion injury. HO is influenced by the nuclear factor erythroid 2-related factor 2 (Nrf2), which has anti-oxidative stress and cytoprotective actions. As found in a study, pre-administration of paeonol could mitigate the degree of liver ischaemia–reperfusion injury by further increasing the expression of Nrf2 mRNA in the cytoplasm of the liver during ischemia–reperfusion and promoting the effect of HO-1 expression, thereby affecting the Nrf2/HO-1 pathway to exert an anti-oxidative stress effect [54]. This suggests that paeonol has a non-negligible role in maintaining the homeostasis of hepatocyte energy metabolism and ultimately in achieving remission of the extent of LIRI injury.

### Alcoholic liver disease

Alcoholic liver disease (ALD) can be described into a clinical disorder of common liver function. And the main causes of ALD are oxidative stress, inflammatory reactions, metabolic disorders, intestinal endotoxins, nutritional imbalance, and inflammatory mediators, which are all induced by alcohol and its derivatives in the metabolic processes directly or indirectly induced [5, 55, 56]. Currently, there are no effective clinical treatments for ALD other than alcohol cessation. Therefore, it is of important practical implications to develop novel agents to prevent and control ALD as a treatment for acute alcoholic liver injury. Among them, the natural product paeonol is an important drug used for the prevention and treatment of ALD in recent studies.

It is well known that the inflammatory response in alcoholic liver injury is thought to be driven by increased alcohol-induced intestinal endotoxin absorption and secretion of pro-inflammatory cytokines and chemokines by activated Kupffer cells [57]. These factors include tumor necrosis factor-α (TNF-α), interleukin-1 (IL-1), interleukin-8 (IL-8), MCP-1 (monocyte chemotactic protein-1), ROS, and NO. As demonstrated by Hu et al. study, paeonol prevented intrahepatic granulocyte mobilization and activation through inhibition of these cytokines and chemokines, thereby attenuating alcoholic liver injury, exhibiting liver fat deposition and reduction of inflammation [58]. To investigate the potential association between antioxidant genes activation and the suppressive capacity of paeonol against alcohol-induced oxidative stress, Sun et al. [59] examined the protein levels of quinone oxidoreductase 1 (NQO-1) and HO-1 in mice before and after paeonol treatment for alcoholic liver injury. The results showed that paeonol pretreatment further antagonized the inhibition of alcohol-induced suppression of silent information regulator sirtuin 1 (SIRT1) expression while upregulating the expression of NQO-1 and HO-1, as well as Nrf2 nuclear translocation. SIRT1 protein is a highly conserved the depletion of nicotinamide adenine dinucleotide (NAD)-dependent deacetylase that plays a role in regulating inflammation [60, 61]. Through this mechanism, paeonol significantly attenuated oxidative stress and inflammation production, thus effectively ameliorating acute alcohol-induced liver injury.

**Fig. 2:**
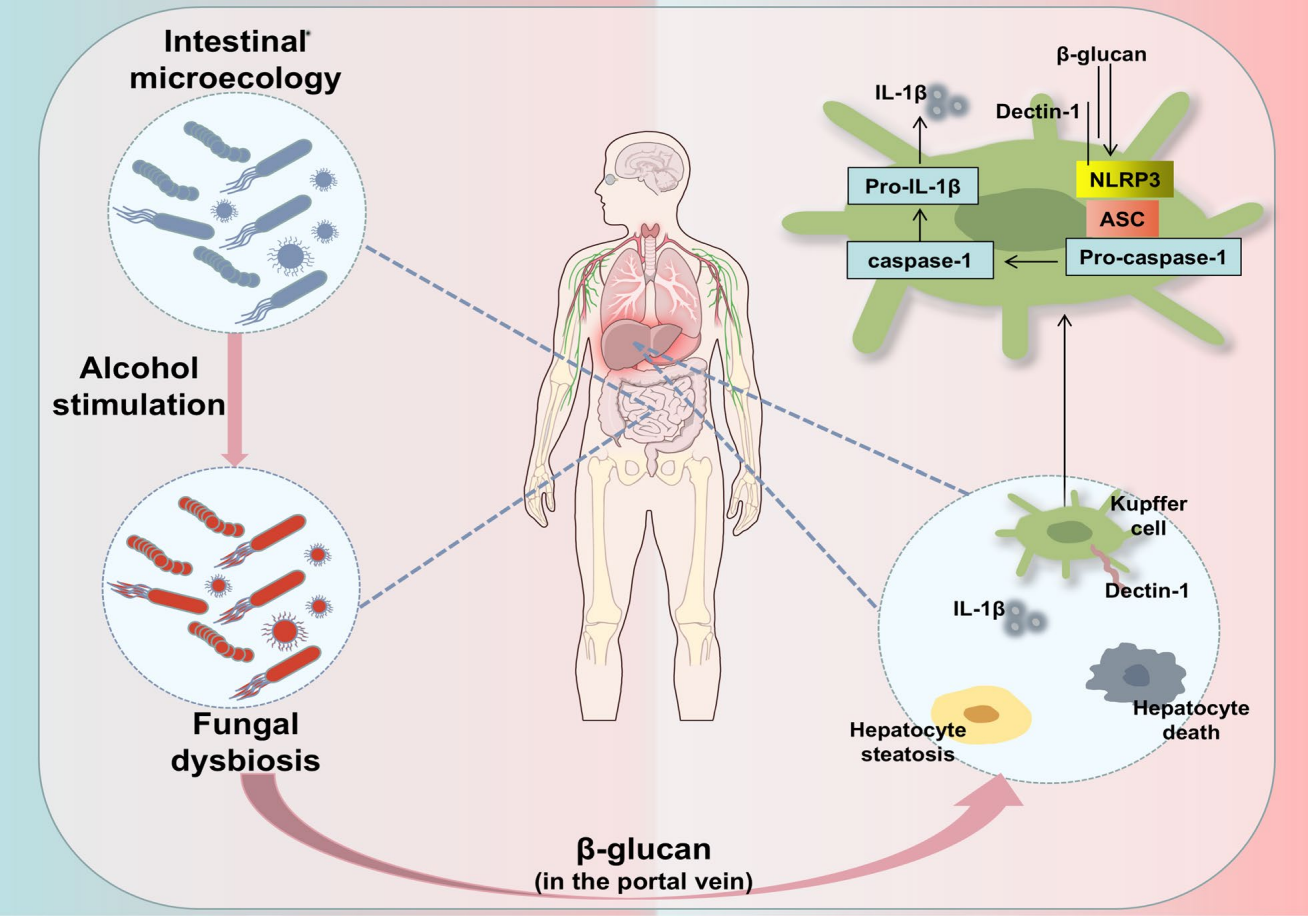
Chronic excessive alcohol consumption leads to dysbiosis of the intestinal fungal flora. One of them, Candida albicans, produces 1,3-β-D glucan that enters the liver and induces Kupffer cells to produce NOD-like receptor family pyrin domain containing 3 (NLRP3) and IL-1β, which leads to the inflammatory response in alcoholic liver disease (ALD)

It has been documented that alcohol and its metabolites could induce the intestinal tract to produce substantial quantities of inducible nitric oxide synthase (iNOS), iNOS activates intracellular non-specific protease C by reacting with microtubule proteins, which increases intercellular permeability [62, 63]. The result is disruption of intestinal barrier function. The relationship between ALD and intestinal flora has also been demonstrated in several studies [64–66]. WU et al. have found that paeonol could attenuate ALD-induced intestinal barrier disorders (Fig. 2), lipid droplet accumulation, and inflammatory lesions in the liver, and has anti-ALD activity [67]. Dendritic cell-associated C-type lectin-1 (Dectin-1) prevents chronic liver disease by inhibiting Toll-like receptor 4 (TLR4) signaling in hepatitis cells and stellate cells [68]. WU et al. in their study also found that paeonol prevented chronic liver disease by modulating 1,3-β-D glucan-mediated Dectin-1/IL-1β axis, suppressing inflammasome-mediated maturation of IL-1β, which inhibited the inflammatory response in ALD and prevented inflammatory injury in the liver. This is another great progress in exploring the mechanism of paeonol in the treatment of ALD.

### Non-alcoholic fatty liver disease

Non-alcoholic fatty liver disease (NAFLD) refers to the presence of steatosis in the liver when secondary causes of fatty liver are excluded, such as excessive alcohol consumption (diagnostic criteria typically set at daily alcohol intake below 20 g for women and below 30 g for men) and other known triggers of fatty liver [69]. NAFLD is common in developing as well as developed countries. The worldwide epidemiological burden of NAFLD has been approximated as approximately 25%, ranging from 13% in Africa to 42% in Southeast Asia [70]. The core mechanism of its pathogenesis lies in a severe imbalance of lipid metabolism in the liver [71]. So far in the clinic the results of drugs for the treatment and prevention of NAFLD have been less than satisfactory. Fortunately, the effect of the natural product paeonol on liver lipids demonstrates its great potential for the treatment of NAFLD.

Yan et al. showed that paeonol ameliorated the symptoms of non-alcoholic steatohepatitis (NASH) by modulating the gut microbial ecology and tryptophan metabolic circuitry [72]. In particular, paeonol reduced a variety of harmful bacteria such as Shigella spp. and Enterobacteriaceae and increased indole-producing probiotics such as Lactobacillus. The aryl hydrocarbon receptor (AhR) is a ligand-activated transcription factor with important functions in physiological and immune responses by binding to a variety of endogenous substances [73, 74]. In response to inflammatory stimuli, ligand-activated AhR prevents excessive induction of pro-inflammatory cytokines by cells such as fibroblasts, endothelial cells, and macrophages [75]. NOD-like receptor family pyrin domain containing 3 (NLRP3) is a cytoplasmic pattern-recognition receptor (PRR), and the nucleotide-binding domain of the leucine-rich repeat family of NLRP3 inflammatory vesicles is required for the host immune system to defend against external infection [76]. After further experiments, Yan et al. also found that paeonol up-regulated the protein expression of AhR in NASH mice and inhibited the expression of downstream inflammatory markers NLRP3 and Caspase-1 genes in the liver. In other words, paeonol also effectively affected the AhR/NLRP3/Caspase-1 inflammatory pathway, which served to inhibit hepatitis in NASH mice. Therefore, we believe that paeonol may be a natural product with great potential for future use in the treatment of NAFLD.

The treatment of NAFLD/NASH remains an unmet medical need, with current strategies focusing on lifestyle interventions and the use of drugs such as pioglitazone [77, 78], which have limited efficacy and potential risks. The mode of action of paeonol is uniquely multifaceted compared to these generalized approaches. Unlike pioglitazone, which primarily improves insulin sensitivity, paeonol simultaneously targets intestinal dysbiosis, gut barrier function, and liver inflammation. This comprehensive mode of action positions paeonol as a potential complementary or foundational multi-targeted therapeutic agent, not necessarily a replacement for existing therapies, which may more holistically address the complex pathogenesis of NAFLD/NASH. Future studies exploring its synergistic effects with existing metabolic drugs are essential.

### Liver fibrosis

Liver fibrosis represents a wound-healing response to liver injury from various causes, an irreversible pathological change in the liver, and a compensatory response to tissue repair of various chronic liver injuries [79]. Epidemiologic evidence shows that 5 percent of deaths in developed countries are due to fibrotic disease [80]. The core mechanism of liver fibrosis involves the transformation of abnormally activated HSCs into myofibroblasts following chronic liver injury [81]. These cells then massively produce and deposit extracellular matrix (ECM), ultimately forming scar tissue [81]. The current anti-liver fibrosis drugs are not effective, so further development of effective and safe anti-liver fibrosis drugs is imperative. Among them, paeonol was demonstrated to have salient anti-liver fibrosis effects in experimental studies.

**Fig. 3:**
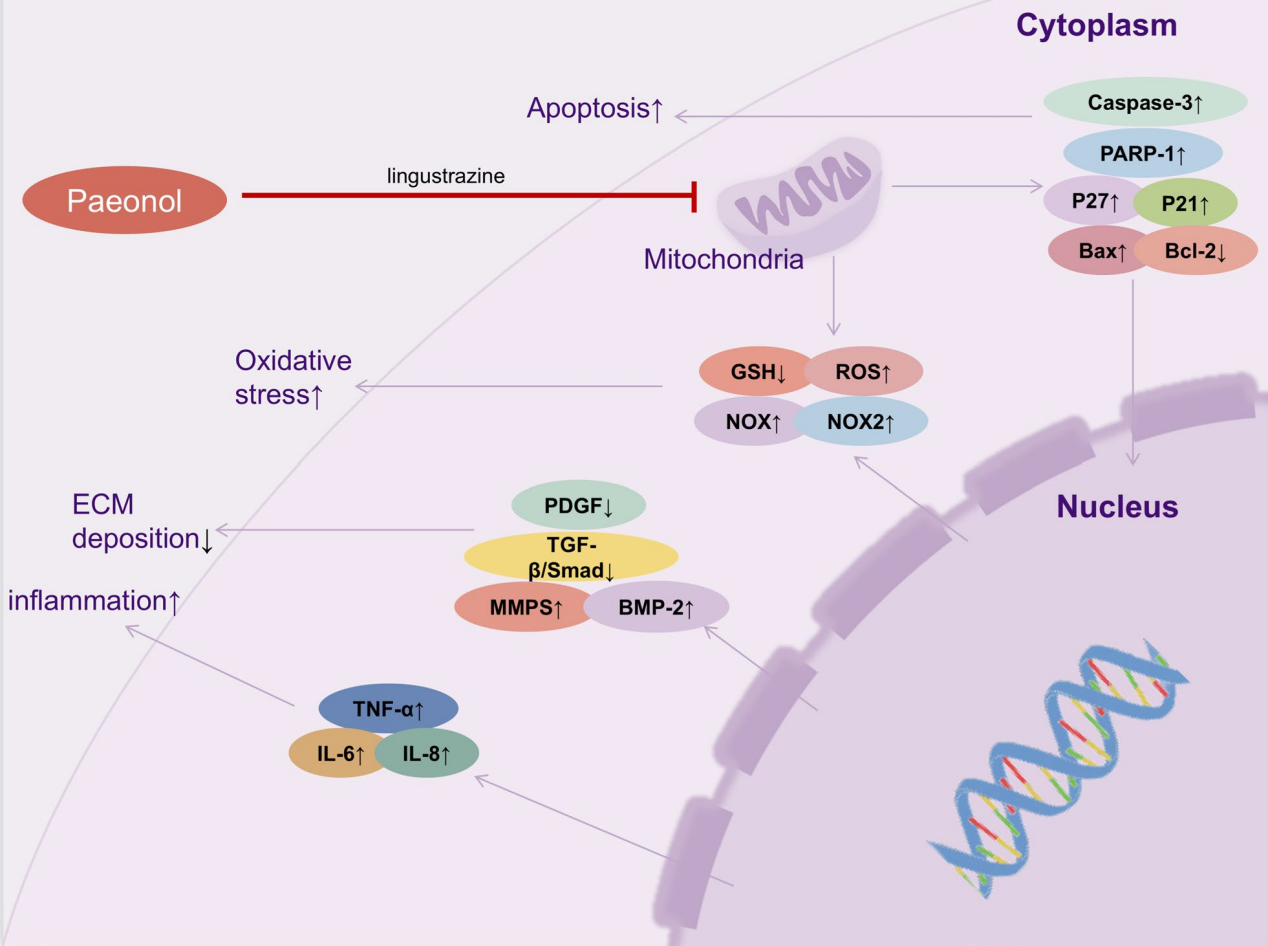
In the case of liver injury, paeonol and ligustrazine combination therapy accomplishes the protective effect of paeonol and ligustrazine on liver function by disrupting the mitochondrial function of hepatic stellate cells (HSCs) and inhibiting oxidative stress, inflammation, apoptosis, and extracellular matrix deposition

It is well known that activated HSCs play a key role in regulating the fibrosis process and can express many marker molecules, such as collagen type I (COL I), α-smooth muscle actin (α-SMA), desmin, and waveform proteins, and connective tissue growth factor (CTGF) is significantly enriched under liver fibrosis conditions [82]. Kong et al. [83] demonstrated that paeonol substantially decrease the protein levels of these marker molecules in a CCl_4_ rat model and interfered with the cell cycle of HSCs in a dose-dependent manner, significantly inhibiting primary HSCs proliferation. Importantly, paeonol had no significant cytotoxic effects. This revealed an important role of paeonol on CCl_4_-induced liver fibrosis in rats. Not coincidentally, another study also found that paeonol significantly alleviated oxidative stress and inflammatory response to prevent CCl_4_-induced liver fibrosis [12]. In addition to this, paeonol can inhibit mitochondrial function as well as act as a potent antioxidant in liver fibrosis. Kong et al. demonstrated that paeonol alleviated oxidative stress, inflammatory responses and induced apoptosis in rat liver fibrotic cells, with potential protective effects against CCl_4_-induced liver fibrotic injury in rats [84] (Fig. 3). In this study, the ATP levels in the mitochondria of HSCs were significantly reduced after paeonol administration, and Cytochrome C (Cyto-C) in the mitochondrial membrane interstitial space was released into the cytoplasm, leading to impaired mitochondrial function in HSCs. In conclusion, paeonol provided ideas for developing new strategies to combat liver fibrosis.

### Hepatocellular carcinoma

Hepatocellular carcinoma (HCC) is defined as a primary tumor originating from hepatocytes and is a widespread outcome of cirrhosis [85]. According to the latest data of statistics, the global incidence of new cases of HCC is 870,000 accounting for 4.3% of new malignant tumors in the world, while the number of deaths of HCC is as high as 760,000. HCC has become the third malignant tumor with the highest mortality rate in the world, which constitutes a great menace to survival and development of the whole mankind [86]. Many factors are associated with the progression of HCC, including hepatotoxins, alcohol consumption, genetically modified hepatitis, and viral infections [87]. In recent years, people have gradually turned their attention to natural products when looking for alternatives to anti-cancer drugs. And paeonol has shown good antitumor activity [88–90]. Therefore, paeonol is considered to be a potentially effective and safe antineoplastic drug that can be used as a substitute or adjunct to traditional cancer treatments.

A study demonstrated that paeonol exerts antitumor effects intumor-bearing mice by inducing tumor cell apoptosis and stimulating interleukin-2 (IL-2) and TNF-αproduction [91]. Furthermore, another study demonstrated that paeonol in combination with cisplatin (CDDP) exerted significant synergistic apoptosis-inducing and growth-inhibitory effects on human  HCC cell lines SMMC-7721 and HepG2 through upregulation of the Bcl-2 family and cell cycle arrest [92]. This study demonstrated that paeonol enhanced the cytotoxicity of CDDP on  HCC cells SMMC-7721 and HepG2, and significantly increased the ability to induce apoptosis. Not coincidentally, Zhang et al. demonstrated that paeonol altered the expression of oncogenes in HCC cell lines, thereby ultimately inhibiting tumor growth [93]. This study showed that paeonol increased the cytotoxicity of 5-fluorouracil (5-FU) on human HCC cells, induced apoptosis and DNA breaks in HCC cells, and inhibited the proliferation of HCC cells. Moreover, it is well known that PTEN is a suppressor gene of tumor, which can inhibit the activation of the oncogene Akt [94]. Among them, Akt1 and Akt2 act as isoenzymes of Akt, and they are both related to tumorigenesis [95]. Zhang et al. also demonstrated that paeonol treatment reduced Akt1 and Akt2 mRNA expression. In conclusion, paeonol can alter the expression of PTEN/Akt in  HCC cells, thus ultimately inhibiting the development of HCC.

Matrix metallopeptidase (MMP), as a gelatinase, is considered one of the most important antitumor targets due to its profound effects in inflammatory processes and tumor progression [96]. In addition, it has been documented that up-regulation of miR-21-5p expression level promotes HCC generation [97], whereas krüppel-like factor 6 (KLF6) shows a downward trend in HCC [98, 99]. Cai et al. demonstrated that paeonol could promote KLF6 expression by down-regulating miR-21-5p expression in human HCC cells and suppressed the oncogenic processes, thereby promoting programmed cell death and modulating hepatocarcinogenesis progression [100]. Studies have shown that the levels of matrix metallopeptidase 2 (MMP2) and matrix metallopeptidase 9 (MMP9) were also inhibited in mice treated with paeonol. At the same time, paeonol also reduced the expression level of miR-21-5p. Similarly, knockdown of miR-21-5p significantly increased the expression of KLF6 in cells. In conclusion, paeonol may regulate the progression of HCC through multiple mechanisms.

Previous reports revealed the relationship between paeonol, survivin and cyclooxygenase-2 (COX-2), for example, paeonol decreased the expression levels of COX-2 and survivin in lung adenocarcinoma cells [101], and also decreased the expression of survivin in ovarian cancer cells [102]. Similar to previous reports, PGE2 significantly increased survivin expression in Huh-7 and HepG2 cells [103]. Liu et al. found that paeonol could target survivin through the COX-2/PGE2 pathway and regulate the changes in survivin levels, thus exerting antitumor effects in HCC exerting antitumor effects on HCC [104]. In this study, PGE2 alone increased survivin levels, but the combination of PGE2 and paeonol decreased survivin expression. In conclusion, paeonol has great potential in the development of therapeutic agents for hepatocellular carcinoma.

## Regulatory mechanism of paeonol in the treatment of liver diseases

With the in-depth study of paeonol, it has been gradually discovered that paeonol can affect the development of some liver diseases by modulating diverse signaling pathways. For instance, in studying the effects of paeonol on hepatotoxicity which caused by EPI, it was showed that paeonol could alleviate EPI-induced hepatotoxicity in mice by regulating the AMPK/mTOR signaling pathway [29]. In addition, it was found that PI3K/Akt signaling pathway, TGF-β/Smad signaling pathway, and NF-κB signaling pathway may also be connected with liver disease. The connection and function of the above signaling pathways in some liver diseases will be described in detail below.

### AMPK/mTOR signaling pathway

**Fig. 4:**
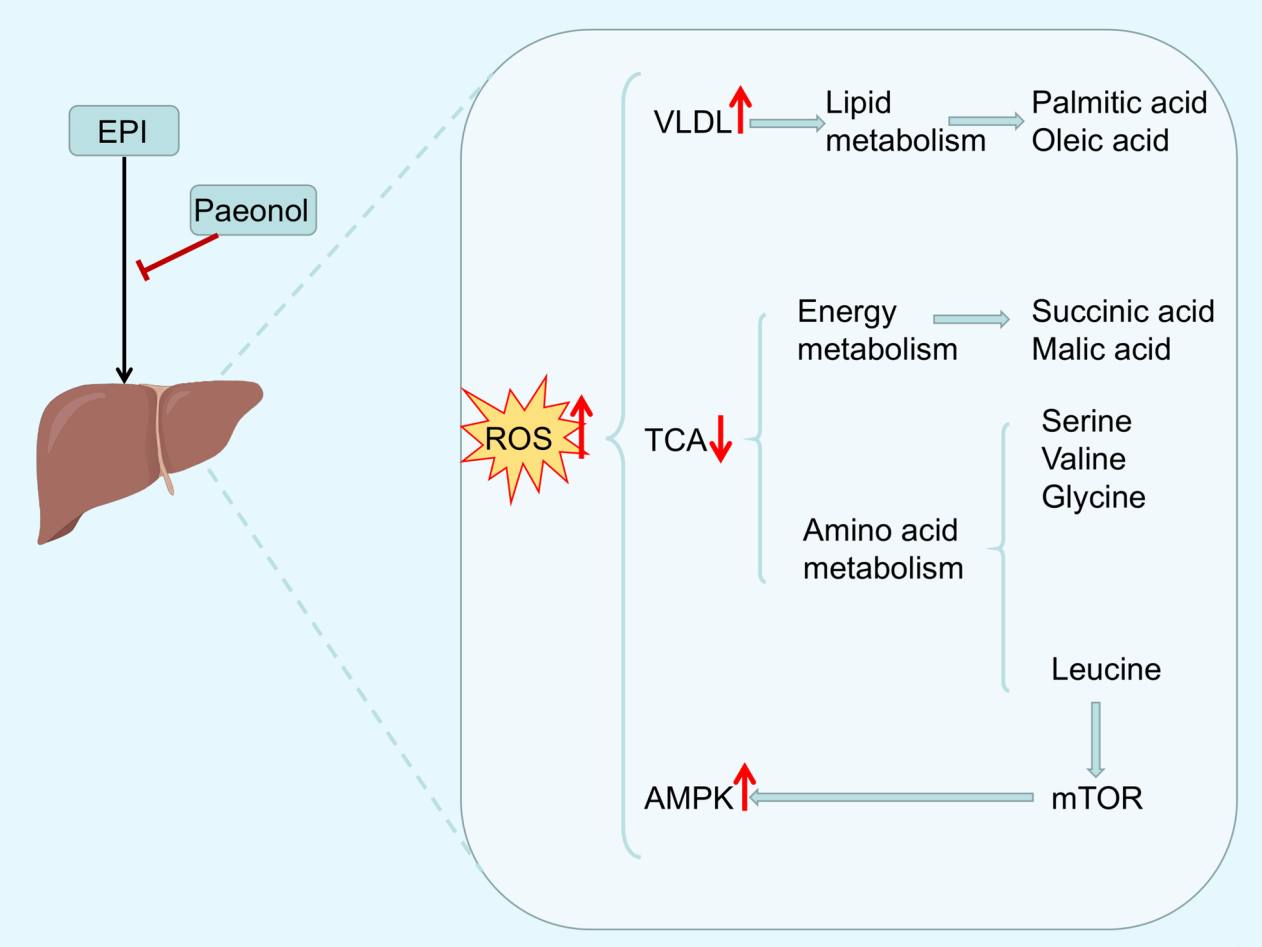
Molecular mechanisms involved in the attenuation of EPI (Epirubicin)-induced hepatotoxicity in mice by paeonol

AMP-activated protein kinase (AMPK) is a heterotrimer composed of an α-, a β and a γ-regulatory subunit [105]. Functioning as the central energy rheostat of eukaryotic cells, AMPK exerts the negative regulation to the mammalian target of rapamycin (mTOR), which in turn regulates the autophagy signaling pathway [106]. Particularly in liver tissues, AMPK activation is physiologically indispensable for the maintenance of energy homeostasis, which has been validated in several studies [107–110]. Notably, paeonol intervention exhibited significant modulation of the AMPK/mTOR signaling pathway in an EPI-induced hepatotoxicity model [29] (Fig. 4). The experimental data showed that paeonol not only restored the EPI-perturbed levels of p-mTOR and p-AMPK in the liver, but also significantly reduced the expression levels of autophagy-related markers. Specifically, paeonol can block the process of hepatocyte injury caused by excessive autophagy by restoring the dynamic balance of AMPK/mTOR signaling pathway, thus exerting its hepatoprotective effect. This provides a new theoretical basis for a deeper understanding of the hepatoprotective mechanism of natural products.

It is noteworthy that the activation of AMPK by paeonol is not completely independent of other factors. It is well known that AMPK is a negative regulator of NF-κB signaling pathway. This idea of modulating the NF-κB signaling pathway through AMPK and thereby improving liver metabolism and inflammation has been confirmed in studies of other hepatoprotective substances. For example, mulberry extract and p-Synephrine have been shown to effectively regulate lipid metabolism and inhibit inflammation in a NAFLD model through activation of the AMPK/PPAR-γ/ NF-κB signaling pathway and the AMPK/NF-κB signaling pathway, respectively [111, 112]. Thus, it appears that paeonol is likely to indirectly inhibit the pro-inflammatory NF-κB signaling pathway through inhibition of mTOR and activation of AMPK and thus forming a synergistic anti-inflammatory and metabolic regulatory network. This cross-talk emphasizes the holistic nature of hepatoprotective mechanism of paeonol.

### PI3K/Akt signaling pathway

The phosphoinositide 3-kinase (PI3K)/ protein kinase B (PKB/Akt) signaling pathway is a core intracellular signaling pathway. It plays a central role in regulating multiple key physiological processes, including cell growth, proliferation, migration, metabolism, and survival [113]. Notably, the nuclear factor-kappa B (NF-κB) is a key downstream effector of this pathway. Mechanistically, PI3K/Akt activation triggers inhibitor of kappa B kinase (IKK), which results in degradation of IκB proteins [114–116]. This molecular cascade promotes nuclear translocation of the p65 subunit, which ultimately facilitates transcriptional activation of anti-apoptotic targets including survivin and Bcl-2 [114–116]. In the EPI-induced DILI model in 4T1 hormonal mice, paeonol exhibited unique signaling regulatory properties [28]. The study showed that paeonol treatment significantly attenuated EPI-induced down-regulation of PI3K, phosphorylated Akt (p-Akt), and phosphorylated NF-κB (p-NF-κB) levels, while total Akt expression remained unchanged. This suggested that paeonol could inhibit activation of the PI3K/Akt signaling pathway without specific inhibitory effects on Akt synthesis. Importantly, this modulatory effect correlates with an attenuation of the NF-κB-mediated inflammatory response, as evidenced by reduced expression of downstream proinflammatory mediators. In conclusion, the PI3K/Akt signaling pathway plays a crucial role in the alleviation of DILI by paeonol. This provides a key theoretical basis for the translational application of paeonol as a DILI control drug.

### TGF-β/Smad signaling pathway

The transforming growth factor-β (TGF-β) constitutes a fundamental signaling pathway for sustaining vital physiological functions and one of the important mediator involved in the fibrosis process [117]. The TGF-β signaling pathway facilitates extracellular matrix deposition primarily through activation of typical Smad-dependent signaling, which ultimately drives tissue scarring through myofibroblast activation and collagen overproduction [118]. Smad2 and Smad3 are further phosphorylated in the nucleus of cells after TGF-β activation are further phosphorylated and translocated to the nucleus as heteromeric complexes that regulate fibroblast activation and extracellular matrix (ECM) production [119], ultimately leading to tissue fibrosis [118]. In many animal models, the development of fibrosis can be inhibited by inhibiting the TGF-β1 signaling pathway [120]. And the TGF-β/Smad signaling pathway has received increasing attention as a potential target for antifibrotic therapy [118]. The TGF-β/Smad signaling pathway was involved in the study of the anti-liver fibrosis effect of paeonol. In the study of Wu et al. the results suggested that paeonol suppressed the excessive activation of TGF-β signaling pathway by downregulating the elevated levels of phosphorylated Smad3 induced by CCl_4_, thus showing the inhibitory effect of the TGF-β/Smad3 signaling pathway to alleviate liver fibrosis caused by CCl_4_ [12]. In addition to this, the combination of paeonol and ligustrazine in the treatment of liver fibrosis was demonstrated to markedly  inhibit the TGF-β/Smad2 signaling pathway. Thus suppressing the onset of collagen deposition, and disrupting the mitochondrial integrity of HSCs to attenuate liver fibrosis [84]. Therefore, it is reasonable to believe that the TGF-β/Smad signaling pathway is a target with great potential for paeonol in the treatment of liver fibrosis.

### NF-κB signaling pathway

As a central regulatory hub of the inflammation, NF-κB serves as a crucial role in the inflammatory cascade by directly regulating the transcriptional activation of immune response-related genes [121, 122]. Its activation involves two signaling pathways. The classical signaling pathway is activated by pro-inflammatory cytokines such as TNF-α, LPS, and IL-1β through an IKKβ-dependent phosphorylation cascade; while, the non-classical signaling pathway depends on IKKα-mediated LTβR/CD40 signaling pathway and specifically induces the formation of p52/RelB heterodimers [123, 124].

**Fig. 5:**
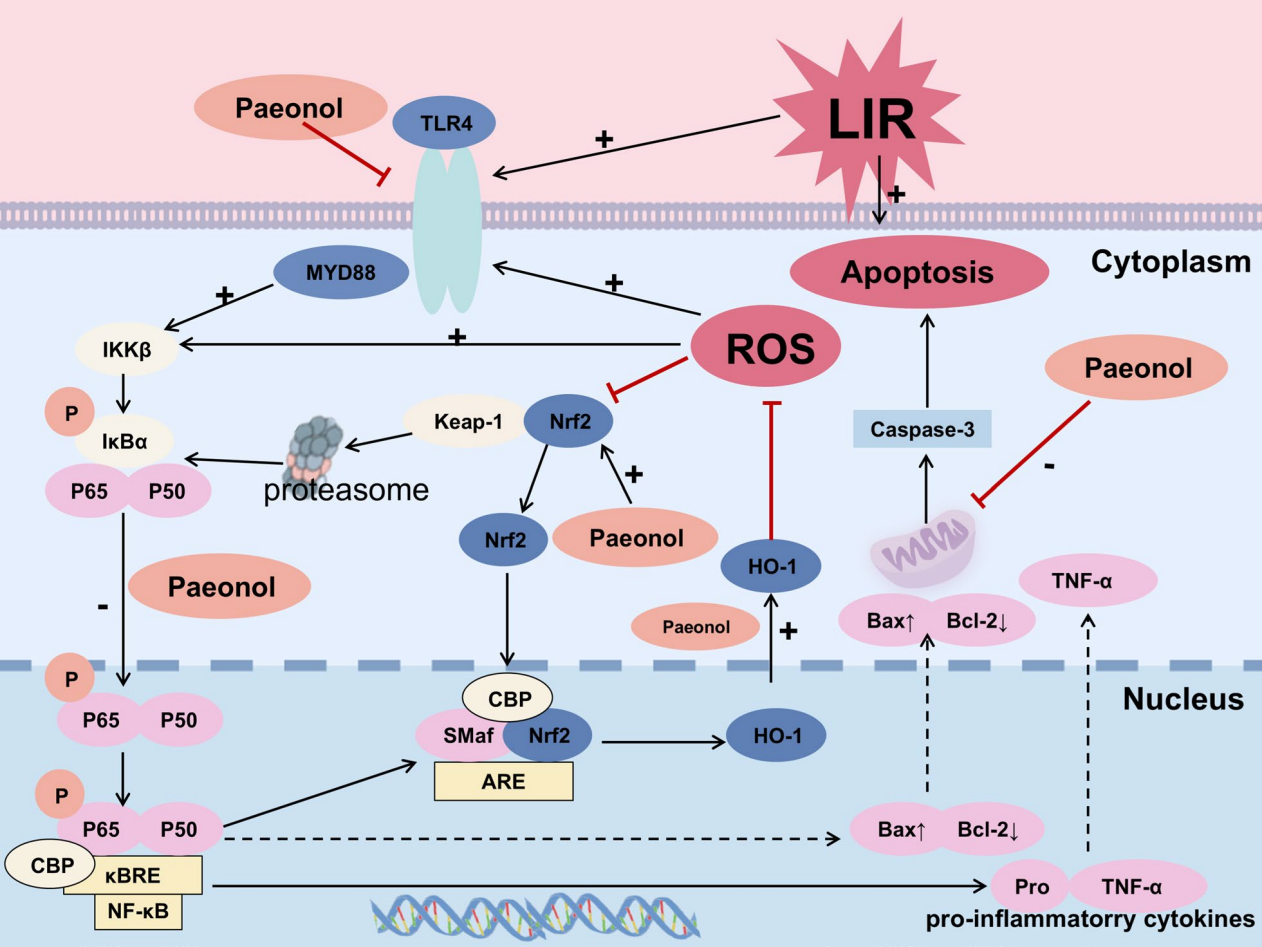
The molecular mechanism by which paeonol attenuates liver ischemia/reperfusion (LIR) in rats

In a DILI model, Xu et al. [13] found that paeonol effectively reversed LPS-induced aberrant phosphorylation of NF-κB and inhibition of nuclear translocation in human normal hepatocytes (L02), confirming that it mitigated DILI by modulating the activation mode of NF-κB. This finding indicated that the hepatoprotective effect of paeonol may be closely associated with its regulation of NF-κB subcellular localization and transcriptional activity. This regulatory effect was further extended in the LIRI model. It was shown that paeonol significantly inhibited the activation of Toll-like receptor 4 (TLR4)/Myeloid differentiation factor 88 (MYD88)/NF-κB signaling axis in liver parenchymal cells and Kupffer cells in the LIRI model, which in turn reduced the release of key mediators of inflammation, such as IL-6, TNF-α [54] (Fig. 5). Among them, TLR4, as a core member of pattern recognition receptors, regulates NF-κB transcriptional activity through dual signaling branches (MYD88-dependent/non-dependent), thereby driving a cascade response of inflammatory cytokines[125, 126]. Paeonol intervention exhibits multi-targeting features, both inhibiting TLR4 receptor activation and blocking the downstream NF-κB nuclear translocation process, which ultimately resulting in the modulation of the inflammatory network.

ALD studies further revealed multiple regulatory mechanisms of paeonol. It was shown that alcohol exposure induced NF-κB (p65) nuclear translocation and accelerated IκB-α protein degradation, while paeonol inhibited NF-κB activation by stabilizing the IκBα/p65 complex [59]. Mechanistic studies suggest that paeonol may indirectly inhibit NF-κB signaling through activation of SIRT1, a NAD + -dependent deacetylase, thereby ameliorating alcohol-induced inflammatory responses and metabolic disorders. Additionally, in a CCl_4_-induced liver fibrosis model [83], paeonol significantly suppressed the phosphorylation of NF-κB (p65) and IκB in liver tissues, blocked the dissociation of IκB/p50/p65 complex, and consequently down-regulated pro-fibrotic gene expression. In vitro experiments confirmed that paeonol completely blocked TNF-α-induced NF-κB (p65) nuclear translocation and inhibited HSCs activation via the mitochondria-dependent apoptotic pathway (Bax/Cyt c/caspase-3 axis). In addition, molecular docking analysis further revealed NF-κB as a direct target of paeonol. It was shown that paeonol could alleviate CCl_4_-induced liver fibrosis by suppressing the NF-κB signaling pathway in HSCs and inducing growth inhibition and mitochondrial apoptosis.

Notably, the antitumor effect of paeonol was similarly associated with NF-κB signaling inhibition in the HCC model. It was shown that paeonol treatment significantly reduced the expression levels of NF-κB p65/p50 heterodimer and phosphorylated API-5, an anti-apoptotic protein, in HCC tissues [127]. Given that NF-κB can transcribe a series of oncogenes related to cell growth and promote tumorigenesis [128, 129], it suggests that the modulation of NF-κB transcriptional activity by paeonol may be a key mechanism by which it inhibits hepatocellular carcinoma progression.

Overall, the NF-κB signaling pathway, as a core target for paeonol to exert hepatoprotective effects, has demonstrated multidimensional regulatory potential in a variety of liver disease models, including DILI, LIRI, ALD, liver fibrosis, and HCC, by regulating pathological processes, such as inflammatory response, apoptosis, and tumorigenesis. The specific mechanisms of action involves direct targeting of NF-κB complex, regulation of upstream TLR4/MYD88 signaling nodes and activation of epigenetic modifiers such as SIRT1. In addition, SIRT1, as a NAD + -dependent deacetylase [60, 61], can deacetylate the p65 subunit of NF-κB, thereby inhibiting its transcriptional activity. This implies that paeonol achieves indirect epigenetic regulation of NF-κB through activation of SIRT1 on the one hand, and direct inhibition of NF-κB on the other hand. This fine regulation through epigenetic modification factors, combined with direct inhibition, constitutes a potent strategy for synergistic inhibition of liver inflammation and oxidative stress. This provides a theoretical basis for the development of novel therapeutic strategies for liver disease based on the NF-κB signaling pathway.

## Future perspectives

In the past, paeonol has been broadly applied to the therapy of various diseases and has received increasing attention from researchers. As reported in many studies, paeonol was applied to the treatment of diverse liver diseases through different mechanisms. However, the low water solubility, low oral bioavailability, poor stability, and rapid metabolic elimination in vivo of paeonol have likewise severely restricted its clinical application [130]. In this part, the future application and research of paeonol will be prospected in light of the latest study results and technologies in relevant fields.

Extracellular vesicles (sEVs), as an attractive natural nanoplatform, have emerged in recent years in the development of novel nanoplatform-based drug delivery systems (DDS). Among them, exosomes, the smallest extracellular vesicles, have received extensive attention as key mediators of intercellular communications. Exosomes are nanoscale (30-150 nm) natural vesicles physiologically released by cells[131]. Their bilipid membrane-based structure allows hydrophilic molecules to enter the aqueous space and hydrophobic drugs to enter the lipid membrane [132, 133]. In addition, the natural origin of exosomes reduces potential toxicity and immunogenicity [134]. These inherent properties make them well suited for use as DDS [135]. For example, in order to avoid the limitations when administering curcumin, researchers developed a natural delivery system that encapsulates curcumin in goat's milk sEVs, which can be used as a nanotherapeutic alternative for experimental chronic liver disease [136]. In this study, the researchers demonstrated that DDS can be used as a nanocarrier for hydrophobic drugs, where the exosomal ginger cumin (sEVCurAc) used as a DDS could be delivered into the liver and ameliorate CCl_4_-generated liver damage, resulting in a reduction of fibrotic deposition and aminotransferase levels [136]. Paeonol and curcumin are both phenolics extracted from natural drugs. From this, it is natural to hypothesize that paeonol can be loaded by corresponding sEVs to solve the existing delivery difficulties of paeonol for better treatment of liver diseases, especially to improve the targeting of paeonol for the treatment of HCC. This enriches our understanding of delivering paeonol with nanocarriers for the treatment of liver diseases and give us new ideas for the therapy.

It is important to note that due to the complexity of the pathogenesis of liver disease, single therapies often fail to achieve optimal results. The excellent safety profile and multi-targeting properties of paeonol make it a promising candidate for combination therapy. Future strategies should focus on combinations that produce synergistic effects through complementary mechanisms of action. For example, in HCC, combining paeonol (which promotes apoptosis by inhibiting PI3K/Akt/NF-κB) with first-line chemotherapeutic agents, such as sorafenib, may improve the antitumor efficacy and potentially overcome the resistance to chemotherapy, thus decreasing the dose of toxic drugs. Beyond that, paeonol has potential for several advanced clinical therapeutic applications. We know that increased adipogenesis from the head has been observed in several cancer types [137], where the expression of major enzymes associated with adipogenesis from the head, including fatty acid synthase(FASN), is increased in tumor lesions [138, 139]. However, evidence is emerging that there are other mechanisms that support hepatocyte proliferation and survival without the proliferation of fat, meaning that deletion of the Fasn gene only delays tumor formation [140]. Because FASN deletion triggers a compensatory activation of the binding protein 2/ hydroxy-3-methyl-glutaryl-coenzyme A reductase (SREBP2/HMGCR) the cascade reaction of metabolism in hepatocellular carcinoma cell cells, this allows triggering a vicarious upward adjustment of HMGCR and cholesterol biosynthesis [140]. Therefore, effective prevention or treatment of HCC may require targeting both FASN and HMGCR to significantly decrease the growth of tumor in HCC patients. Therefore, based on current research progress, we can anticipate designing paeonol as a dual-target modulator for improved treatment of liver diseases. Similarly, inspired by the “inhibition-degradation” bifunctional molecule YB-3-17 (the inhibitor of mTOR) [141], paeonol can be converted into a chimeric molecule that simultaneously inhibits the NF-κB signaling pathway and degrades a key pro-fibrotic protein. This strategy is expected to provide long-lasting inhibition of liver fibrosis through a catalytic degradation mode, while reducing off-target effects of conventional small molecule drugs.

In conclusion, the future development of paeonol is undoubtedly a promising research direction, which requires the integration of multidisciplinary cross-cutting technologies such as metabolic regulation, intelligent delivery and molecular design. Through interdisciplinary technological innovation, paeonol is expected to be upgraded from a traditional monomer drug to a core component of an intelligent therapeutic system. It will open up new pathways in the fields of precise intervention, drug resistance overcoming and tissue regeneration in liver diseases. Ultimately, it will promote the innovative application of natural active ingredients in modern medicine.

## Conclusion

As a natural product, paeonol is involved in a variety of pharmacological effects, including anti-inflammatory, neuroprotective, anti-tumor, anti-cardiovascular disease, regulation of glycolipid metabolism, and analgesic effects. But paeonol preparations are still not much used in the clinic. At present, paeonol preparations are commonly used in allergic rhinitis, allergic purpura, systemic lupus erythematosus, allergic skin diseases, rheumatic arthritis and rheumatoid arthritis (Table 2). In addition to this, researchers have been discovering the powerful role it plays in liver diseases. In the field of liver diseases, paeonol demonstrates broad-spectrum therapeutic potential across multiple pathological stages, including DILI, LIRI, ALD, NAFLD, liver fibrosis, and HCC.

**Table 2:** Progress in the clinical application of paeonol

Name of clinical trial	Year of commencement of the trial	Indications	Clinical phase	Applicant Organization	Country	Registration numbers
Phase II Clinical Trial of Safflower paeonol cream	2014	Swelling and pain from soft tissue injuries and osteoarthrosis	Phase II	Guangdong Zhongda Nanhai Marine Biotechnology Engineering Center Co	China	CTR20131770
Human tolerance of saffron paeonol cream in phase I clinic	2014	Swelling and pain from soft tissue injuries and osteoarthrosis	Phase I	Guangdong Zhongda Nanhai Marine Biotechnology Engineering Center Co	China	CTR20131700

Recent studies have indicated that gut microbiota is closely linked to liver health, profoundly influencing the onset and progression of various liver diseases through the “gut-liver axis.” Whether in ALD or NAFLD, acute liver injury, liver fibrosis, or even HCC, gut dysbiosis and intestinal barrier disruption are commonly observed. These conditions facilitate bacterial translocation and the migration of harmful microbial metabolites to the liver, triggering inflammation, immune responses, and fibrosis [142]. By reshaping the microbial ecosystem [143, 144], regulating microbial metabolites [145, 146], and reversing cross-domain dysbiosis [147] can effectively improve liver injury, slow disease progression, and enhance therapeutic outcomes. This approach opens highly promising new avenues for the prevention and treatment of liver diseases. Notably, the therapeutic effects of paeonol transcend traditional multi-target perspectives by exerting its benefits through effective regulation of the gut-liver axis. As demonstrated in ALD and NASH models [67, 72], paeonol alleviates liver disease by correcting dysbiosis, enriching beneficial microbiota, and strengthening the intestinal barrier. This mechanism aligns closely with cutting-edge research on gut microbiota modulation for liver disease treatment, highlighting the immense potential value of paeonol as a modern therapeutic agent.

Despite its promising pharmacology, the inherent pharmacokinetic limitations of paeonol (including poor water solubility, low oral bioavailability, and rapid systemic clearance) significantly hinder its clinical translation. To realize the therapeutic potential of paeonol, it is necessary to identify suitable methods to enhance its delivery and efficacy. The most promising avenue lies in advanced drug delivery systems (DDS). Among these, exosomes emerge as a particularly promising drug-carrying platform due to their superior biocompatibility, passive targeting capabilities, and potential for active liver-specific targeting. The successful application of such DDSs is crucial for advancing paeonol from a preclinical candidate to a clinically viable therapeutic. In conclusion, while paeonol is a highly promising natural product for liver disease therapy, the development of efficient targeted delivery systems is not optional but essential to unlock its full clinical potential.

## Data Availability

No data was used for the research described in the article.
